# Vitamin B6 in nausea and vomiting of pregnancy: metabolism, mechanisms, and clinical implications—a narrative review

**DOI:** 10.3389/fnut.2026.1836915

**Published:** 2026-08-06

**Authors:** Zhuwei Gao, Yu Yao, Hui Chang, Yu Wang, JianNan Yu, BaiChao Shi, Yue Gao, XiaoKe Wu, JingShu Gao

**Affiliations:** 1 Graduate School, Heilongjiang University of Chinese Medicine, Harbin, Heilongjiang, China; 2 Department of Gynecology, First Affiliated Hospital, Heilongjiang University of Chinese Medicine, Harbin, Heilongjiang, China; 3 Zhejiang Provincial Hospital of Chinese Medicine, and Zhejiang Chinese Medical University, Hangzhou, Zhejiang, China

**Keywords:** clinical studies, hyperemesis gravidarum, mechanisms, nausea and vomiting in pregnancy, pyridoxal-5′-phosphate, vitamin B6

## Abstract

Nausea and vomiting of pregnancy (NVP), including its severe form, hyperemesis gravidarum (HG), is a common condition during early pregnancy, with complex and incompletely understood pathophysiological mechanisms. In recent years, attention has increased toward the potential role of vitamin B6 in the management of NVP. This narrative review aims to summarize and integrate current evidence on vitamin B6 metabolism, pregnancy-related physiological changes, potential mechanisms of action, clinical efficacy, safety, and relevant international guideline recommendations in NVP. Current evidence generally supports vitamin B6 as a first-line foundational treatment for mild-to-moderate NVP, particularly when combined with doxylamine, demonstrating favorable clinical utility. Emerging evidence suggests that vitamin B6 may contribute to the development and regulation of NVP through multiple pathways, including neurotransmitter metabolism, neuroendocrine regulation, and mechanisms of the gut-brain axis. However, direct clinical evidence supporting these mechanistic links remains limited. Future studies integrating biomarkers, neuroendocrine pathways, and high-quality clinical investigations are warranted to further clarify the mechanistic role and clinical value of vitamin B6 in NVP.

## 1 Introduction

Nausea and vomiting of pregnancy (NVP), a common pregnancy disorder characterized by nausea and vomiting predominantly during the first trimester, affects approximately 70–80% of pregnant women, whereas hyperemesis gravidarum (HG), its severe form, affects 0.3–2% of pregnancies (1). Symptoms usually begin between 4 and 7 weeks of gestation. They often peak around 9 weeks and improve before 20 weeks in most cases (2). In some patients, symptoms are more severe and may persist. Persistent nausea and vomiting can reduce quality of life. They may also lead to dehydration, electrolyte imbalance, malnutrition, and weight loss. In severe cases, the risk of adverse pregnancy outcomes may increase (2). Given these potential clinical consequences, standardized assessment of NVP severity is important in clinical research. The Pregnancy-Unique Quantification of Emesis and Nausea (PUQE) score is a validated tool commonly used in pregnancy to assess nausea duration, vomiting frequency, and retching frequency, and its 24-h version (PUQE-24) assesses symptoms over the preceding 24 h (3). Accurate assessment of symptom severity also provides a basis for investigating how clinical manifestations relate to underlying biological mechanisms. In recent years, research on NVP has gradually expanded beyond isolated abnormalities in single hormones or neurotransmitters to encompass multiple dimensions, including placenta-derived factors, neuroendocrine regulation, energy metabolism, and the gut microbiota. However, the potential interactions among these mechanisms and their relationships with clinical symptom severity remain insufficiently understood and require further integration.

Vitamin B6 is a water-soluble vitamin, and its active form is pyridoxal-5′-phosphate (PLP). PLP functions as a coenzyme in many enzymatic reactions (4). Vitamin B6, recommended by international guidelines as a first-line treatment for NVP, is commonly administered as pyridoxine 10–25 mg orally three to four times daily, either alone or in combination with doxylamine when symptoms persist (5). However, existing studies and reviews have primarily focused on the clinical efficacy, safety, or individual mechanistic aspects of vitamin B6. In contrast, a comprehensive integration of pregnancy-related pharmacokinetic changes, their potential interactions with neuroendocrine regulatory mechanisms involved in NVP, and how these mechanisms may relate to clinical symptom improvement remains lacking.

**Figure 1:**
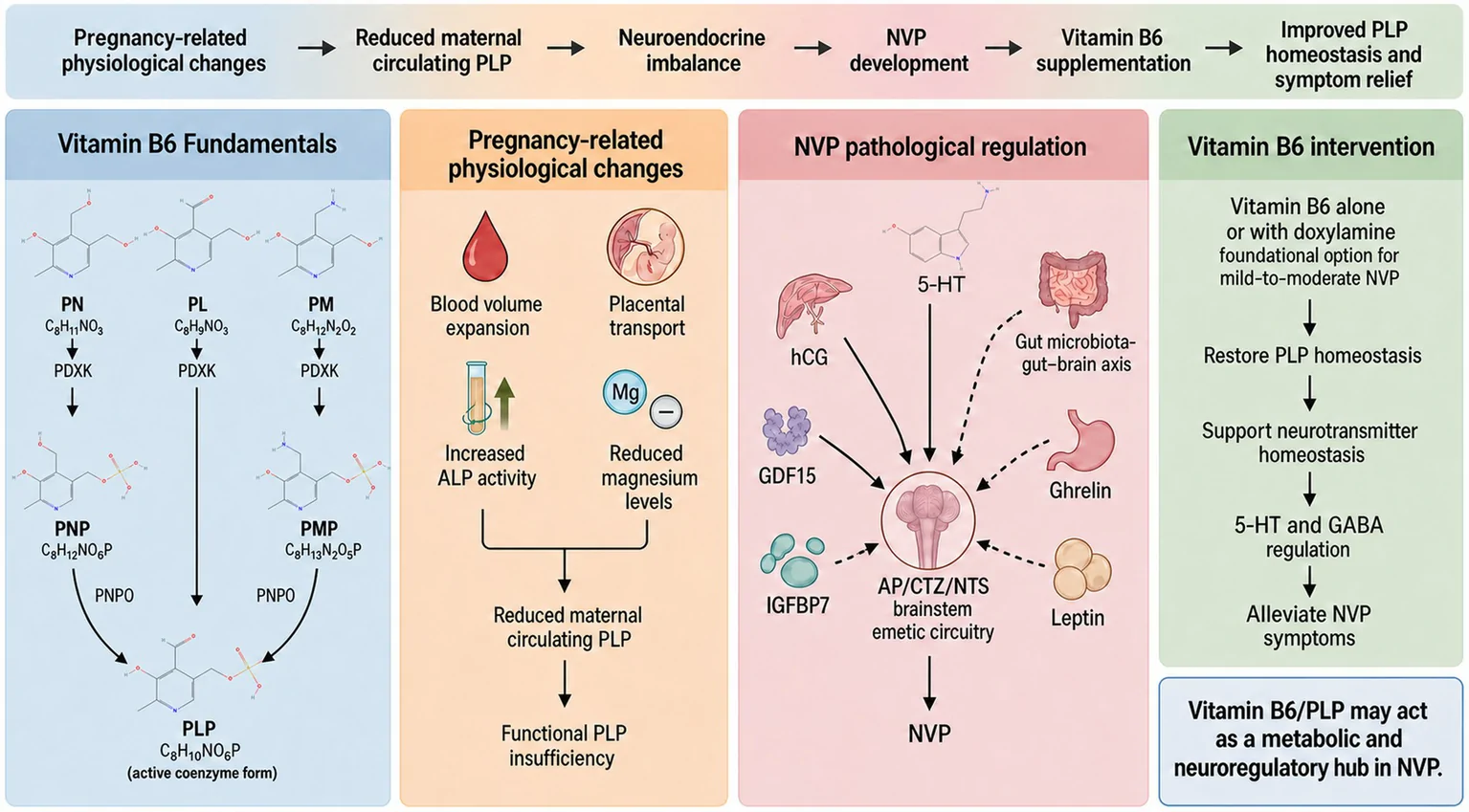
Role and mechanisms of Vitamin B6 in NVP. This graphical abstract summarizes the potential role of vitamin B6 and PLP in NVP, linking pregnancy-related physiological changes, altered maternal vitamin B6/PLP status, potential functional PLP insufficiency, neuroendocrine dysregulation, NVP development, and the potential therapeutic effects of vitamin B6 supplementation. Solid arrows indicate relatively established pathways, whereas dashed arrows indicate potential, indirect, or emerging pathways. PN, pyridoxine; PL, pyridoxal; PM, pyridoxamine; PNP, pyridoxine 5′-phosphate; PLP, pyridoxal-5′-phosphate; PMP, pyridoxamine 5′-phosphate; PDXK, pyridoxal kinase; PNPO, pyridoxine phosphate oxidase; ALP, alkaline phosphatase; Mg, magnesium; 5-HT, 5-hydroxytryptamine; GDF15, growth differentiation factor 15; hCG, human chorionic gonadotropin; IGFBP7, insulin-like growth factor binding protein 7; AP, area postrema; CTZ, chemoreceptor trigger zone; NTS, nucleus tractus solitarius; GABA, *γ*-aminobutyric acid.

Against this background, the present review begins with the fundamental biological characteristics of vitamin B6 and summarizes its dietary sources, bioavailability, absorption, transport, and metabolic regulation, with particular emphasis on pregnancy-related pharmacokinetic changes. In addition, this review further integrates neuroendocrine regulatory mechanisms associated with NVP, clinical evidence, and potential mechanistic pathways to explore the possible links between vitamin B6 metabolism and neurotransmitter regulation, placenta-derived factors, appetite- and energy metabolism-related hormones, as well as the gut-brain axis. By doing so, this review aims to provide a more comprehensive and integrative perspective on the role of vitamin B6 in NVP and to offer insights for future clinical research and individualized intervention strategies (see Figure 1).

## 2 Methodology

This narrative review was informed by a broad literature search of PubMed, Web of Science, and Google Scholar. Literature published primarily between 2015 and 2026 was considered, while earlier foundational studies were included when necessary to provide historical or mechanistic context. Search terms included “vitamin B6,” “pyridoxine,” “pyridoxal-5′-phosphate,” “nausea and vomiting of pregnancy,” “hyperemesis gravidarum,” and terms related to relevant neuroendocrine and gut–brain pathways. Reference lists of relevant articles were also examined to identify additional publications. Literature was selected on the basis of its relevance to the scope of this narrative review; no formal systematic-review protocol, quantitative synthesis, or risk-of-bias assessment was performed.

## 3 Fundamental characteristics and metabolic regulation of vitamin B6

Vitamin B6 is not a single compound but rather a collective term for a group of structurally related vitamers, including the free forms of pyridoxine (PN), pyridoxal (PL), pyridoxamine (PM), and their corresponding phosphorylated derivatives. In addition to serving as a natural antioxidant that reduces advanced glycation end products, maintaining its metabolic homeostasis is closely associated with the development and progression of multiple diseases, including diabetes, cardiovascular disease, and cancer, as well as the prognosis of coronavirus disease 2019 (6). Since its discovery by György and colleagues in 1934, it has been recognized as a cofactor in more than 140 intracellular biochemical reactions (7). This section summarizes the general biochemical forms and core metabolic pathways of vitamin B6 as background for understanding its potential relevance to pregnancy and NVP. Pregnancy-related changes in vitamin B6 status are discussed subsequently.

### 3.1 Core biological characteristics

The six vitamers of vitamin B6 share a common 2-methyl-3-hydroxypyridine backbone and differ only in the substituents at the C4 and C5 positions. They can be broadly classified into free forms and phosphorylated forms. The free forms include PN, PL, and PM, whereas the phosphorylated forms include pyridoxine 5′-phosphate (PNP), PLP, and pyridoxamine 5′-phosphate (PMP). PLP is the principal biologically active coenzyme form of vitamin B6. In studies of non-pregnant populations, PLP has been reported as the predominant circulating vitamin B6 vitamer, accounting for approximately 70–90% of plasma vitamin B6 forms (8); however, pregnancy-specific estimates remain limited. Other vitamin B6 vitamers can be converted to PLP before participating in PLP-dependent physiological functions. Specifically, pyridoxal kinase (PDXK) first catalyzes the phosphorylation of the free forms PN, PL, and PM into their corresponding phosphorylated derivatives, after which pyridoxine phosphate oxidase (PNPO) further oxidizes PNP and PMP to generate PLP (9).

PLP acts as a cofactor in more than 160 enzyme reactions. These reactions include amino acid transamination, decarboxylation, and racemization (8). In the transsulfuration pathway, PLP-dependent enzymes such as cystathionine *β*-synthase and cystathionine *γ*-lyase convert homocysteine into cysteine. This process provides about 50% of the cysteine used for glutathione synthesis. It also links one-carbon metabolism with the antioxidant system (10). In the central nervous system, PLP is involved in amino acid and neurotransmitter metabolism and supports neurotransmitter synthesis and neural activity (11). *γ*-aminobutyric acid (GABA) is the main inhibitory neurotransmitter in the brain, and its synthesis depends on the PLP-dependent enzyme glutamate decarboxylase (GAD), which converts glutamate into GABA. Through this pathway, PLP helps maintain the balance between GABA and glutamate and regulate neuronal excitability; PLP deficiency can markedly reduce GABA production and lead to abnormal neuronal hyperexcitability (12). In addition, vitamin B6 deficiency impairs immune regulation by reducing lymphocyte proliferation and T-cell cytotoxicity, whereas supplementation can restore lymphocyte function and improve immune responses (9).

### 3.2 Dietary sources and factors influencing bioavailability

#### 3.2.1 Major dietary sources

Dietary sources of vitamin B6 are highly diverse and include animal-derived foods, such as meat, poultry, fish, liver, and eggs, as well as plant-derived foods, such as whole-grain products, vegetables, and legumes (13). Animal-derived foods mainly provide vitamin B6 as PL and PM, which are more readily absorbed and converted to PLP in the human body. In contrast, plant-derived foods, including whole-grain products and vegetables, mainly contain PN and its glucoside derivative (PNG) (14). Among plant-derived foods, legumes are important dietary sources of B vitamins. *In vitro* digestion studies further indicate that the bioaccessibility of vitamin B6 vitamers in dried and canned legumes is influenced by vitamer form and the food matrix (15).

#### 3.2.2 External regulators of bioavailability

Different forms of vitamin B6 differ in their absorption and conversion efficiency. Non-phosphorylated forms are absorbed through passive and facilitated diffusion and are subsequently converted intracellularly into active coenzyme forms (16).

The food matrix is an important determinant of vitamin B6 bioavailability. The absorption efficiency of vitamin B6 from plant-derived foods is generally lower than that from animal-derived foods, a difference that may be related to limited glucoside hydrolysis and metabolic conversion mediated by the gut microbiota (17). Therefore, even when dietary intake is adequate, a predominance of plant-derived sources of vitamin B6 may still influence nutritional status. Food processing and cooking methods can also affect vitamin B6 content and bioavailability. As a water-soluble vitamin, vitamin B6 is prone to loss during heating, particularly in the less heat-stable PL and PM forms. In some legumes, *in vitro* digestion models suggest that moderate cooking may increase vitamin B6 bioaccessibility by altering the food matrix and releasing vitamin B6 from bound forms (15).

The gut microbiota may also affect vitamin B6 availability. Studies in Drosophila have shown that intestinal symbiotic bacteria can support host development under nutrient-restricted conditions by synthesizing vitamin B6 (18). This suggests that microbiota-derived vitamin B6 may contribute to host physiological function. Some probiotic bacteria, such as Lactobacillus, can synthesize PMP, a vitamin B6 form with antimicrobial activity that may inhibit several pathogens and contribute to host–microbe interactions and microbial community regulation (19). Other factors may also influence vitamin B6 absorption and utilization. Dietary fiber and antinutritional components, such as phytates, may slightly reduce bioavailability. This may occur through nutrient binding or faster intestinal transit.

In contrast, protein intake may improve vitamin B6 absorption. It may enhance amino acid transport and promote the uptake of forms such as PL (20). Patients with inflammatory bowel diseases, such as Crohn’s disease, are more likely to have vitamin B6 deficiency. This may be related to impaired intestinal absorption and changes in gut microbiota composition (21). Pregnancy-related changes in vitamin B6 status and requirements are discussed separately in the following section.

### 3.3 *In vivo* absorption, transport, and homeostatic regulation

Vitamin B6 is mainly present in serum as PLP. Other circulating forms include PL and 4-pyridoxic acid (PA). Serum PLP concentration is commonly used as a biomarker of vitamin B6 nutritional status (22). Dietary phosphorylated forms of vitamin B6 must first undergo dephosphorylation by alkaline phosphatases on the intestinal mucosal surface before absorption. After conversion to their free forms, they can be absorbed by the small intestine and subsequently participate in systemic transport and metabolic processes (10). PLP cannot cross the cell membrane directly. It must be converted into free PL before entering the portal circulation. In the bloodstream, PL is mainly taken up by the liver through passive diffusion. It is then rephosphorylated by PLK to generate PLP. Unbound PLP can be converted back to PL. A portion is further oxidized to PA and excreted in urine. This is one of the main elimination pathways for vitamin B6 (23). The body does not have a specific storage pool for vitamin B6. Its homeostasis depends on the balance between transport, rephosphorylation, and excretion. In addition, the hydrolysis efficiency of PNG in plant foods, together with individual differences in vitamin B6 conversion capacity, may affect its absorption and utilization (24).

### 3.4 Metabolic conversion pathways and regulatory mechanisms

The liver is a key organ for maintaining vitamin B6 homeostasis and generating PLP, the principal biologically active coenzyme form. Newly generated PLP is released into the bloodstream mainly in an albumin-bound form and participates in a wide range of PLP-dependent biochemical reactions (7, 25). At the molecular level, PLP generation *in vivo* mainly depends on the salvage pathway, in which PDXK phosphorylates free vitamers such as PN, PL, and PM, and PNPO subsequently oxidizes the phosphorylated intermediates to generate PLP. Among these enzymes, PDXK is considered a key regulatory enzyme in the conversion of PL to PLP (26).

## 4 Vitamin B6 status and physiological relevance during pregnancy

During pregnancy, adequate vitamin B6 is essential for maternal physiological health and fetal growth and development. Studies have shown that supplementation with pyridoxine or its active forms can alleviate symptoms of NVP (27); vitamin B6 status also influences maternal glucose homeostasis and may reduce the risk of gestational diabetes mellitus (28). In addition, a deficiency of vitamin B6 can impair heme synthesis, aggravate gestational anemia (29), and may also contribute to pregnancy maintenance by regulating the placental enzyme diamine oxidase (30). Adequate vitamin B6 intake during pregnancy supports fetal growth and genomic stability and helps reduce lipid peroxidation (31). It also supports fetal brain and nervous system development (32). In addition, it may help reduce the risk of cleft lip and palate, neural tube defects (33), and neonatal seizures. It may also lower the risk of low birth weight (34).

### 4.1 Plasma volume expansion and changes in circulating PLP

During pregnancy, blood volume increases by about 40–50% compared to the non-pregnant state. This includes an increase of approximately 1,300 mL in plasma volume and 400 mL in red blood cell volume (35). Although plasma volume expansion may influence circulating biomarker concentrations, reduced maternal PLP levels during pregnancy should not be attributed to hemodilution alone.

One study reported that maternal plasma PLP concentrations tend to decrease progressively throughout pregnancy and are significantly associated with indicators of PLP-dependent transsulfuration function, such as cystathionine concentration (36). This finding suggests that pregnancy may be accompanied by a metabolic state of “functional vitamin B6 insufficiency.”

A large longitudinal study similarly reported marked changes in PLP levels and related functional metabolic indicators during pregnancy, such as the HK ratio and the cystathionine/cysteine ratio (37). In that study, these alterations could not be fully explained by plasma volume expansion alone and were more likely related to increased metabolic demand, altered tissue uptake, and changes in the inflammatory and hormonal environments. In addition, circulating PLP levels begin to decline in early pregnancy and decrease further in late pregnancy. In contrast, the homocysteine/kynurenine ratio and the cystathionine/cysteine ratio rise sharply from early to late gestation, suggesting reduced activity of vitamin B6-dependent metabolic pathways and insufficient PLP levels (37, 38). Vitamin B6 supplementation can improve PLP status, which generally returns to pre-pregnancy levels by 6 months postpartum. Nevertheless, current evidence regarding pregnancy-related changes in vitamin B6 status is largely derived from observational studies, and the relative contributions of plasma volume expansion, altered placental transport, and metabolic adaptation remain incompletely understood. Therefore, the mechanisms underlying pregnancy-associated functional PLP insufficiency warrant further investigation.

### 4.2 Placental transfer and maternal-fetal allocation of vitamin B6

The placenta is the primary interface for maternal-fetal nutrient transport (39). The detection of vitamin B6 in placental extracts suggests that vitamin B6-related metabolic processes occur in placental tissue and may contribute to the regulation of maternal-fetal vitamin B6 transfer (40). Circulating PLP-albumin complexes dissociate after entering the intervillous space through the uterine spiral arteries, and the released free PLP is dephosphorylated by membrane-bound alkaline phosphatase on the trophoblast cell surface to form PL, which can cross the membrane (8, 41). *In vitro* placental perfusion experiments have shown that PL generated by PLP dephosphorylation can be transported bidirectionally between the mother and fetus; however, the transport efficiency from mother to fetus is 2.6 times greater than that from fetus to mother, and the transport concentration and rate of PL show a linear relationship without saturation (42). These findings are more consistent with passive diffusion and challenge the hypothesis of vitamin B6 active transport. In a prospective cohort study, maternal plasma PLP concentrations throughout pregnancy were positively correlated with cord blood PLP concentrations, and at delivery, cord plasma PLP levels were approximately 5 times higher than maternal plasma PLP levels (43). This finding suggests that vitamin B6 may undergo preferential maternal-fetal transfer to meet fetal demands. The placenta expresses pyridoxal kinase, which phosphorylates transported PL into PLP, and available evidence suggests that its activity is maintained across gestation (44). This enzyme plays a fundamental role in maternal-fetal vitamin B6 metabolism. Although the precise mechanism of placental vitamin B6 transport remains unclear, whether mediated through active transport or passive diffusion, current evidence suggests a preferential maternal–fetal allocation of vitamin B6 during pregnancy, which may contribute to reduced maternal circulating PLP concentrations.

### 4.3 Synergistic effects of other physiological factors

Other physiological factors may also affect vitamin B6 levels during pregnancy.

During gestation, maternal basal metabolism increases. At the same time, demand for magnesium rises, and magnesium levels often decrease (45). Magnesium deficiency can influence neurotransmitter release. It can also reduce ATP production, thereby lowering PDXK activity and affecting vitamin B6 metabolism (46).

In addition, placental alkaline phosphatase is released into the maternal circulation during pregnancy. This enzyme can convert PLP into PL, which may reduce vitamin B6 levels (30). PLP bound to albumin is relatively stable and less likely to be degraded (47). Furthermore, previous studies have suggested that pregnancy-related changes in plasma volume and albumin binding may reduce the proportion of albumin-bound PLP, increasing the fraction of free PLP that is more susceptible to alkaline phosphatase-mediated degradation and may contribute to lower circulating PLP levels (47, 48).

## 5 Neuroendocrine regulatory mechanisms of NVP

**Figure 2:**
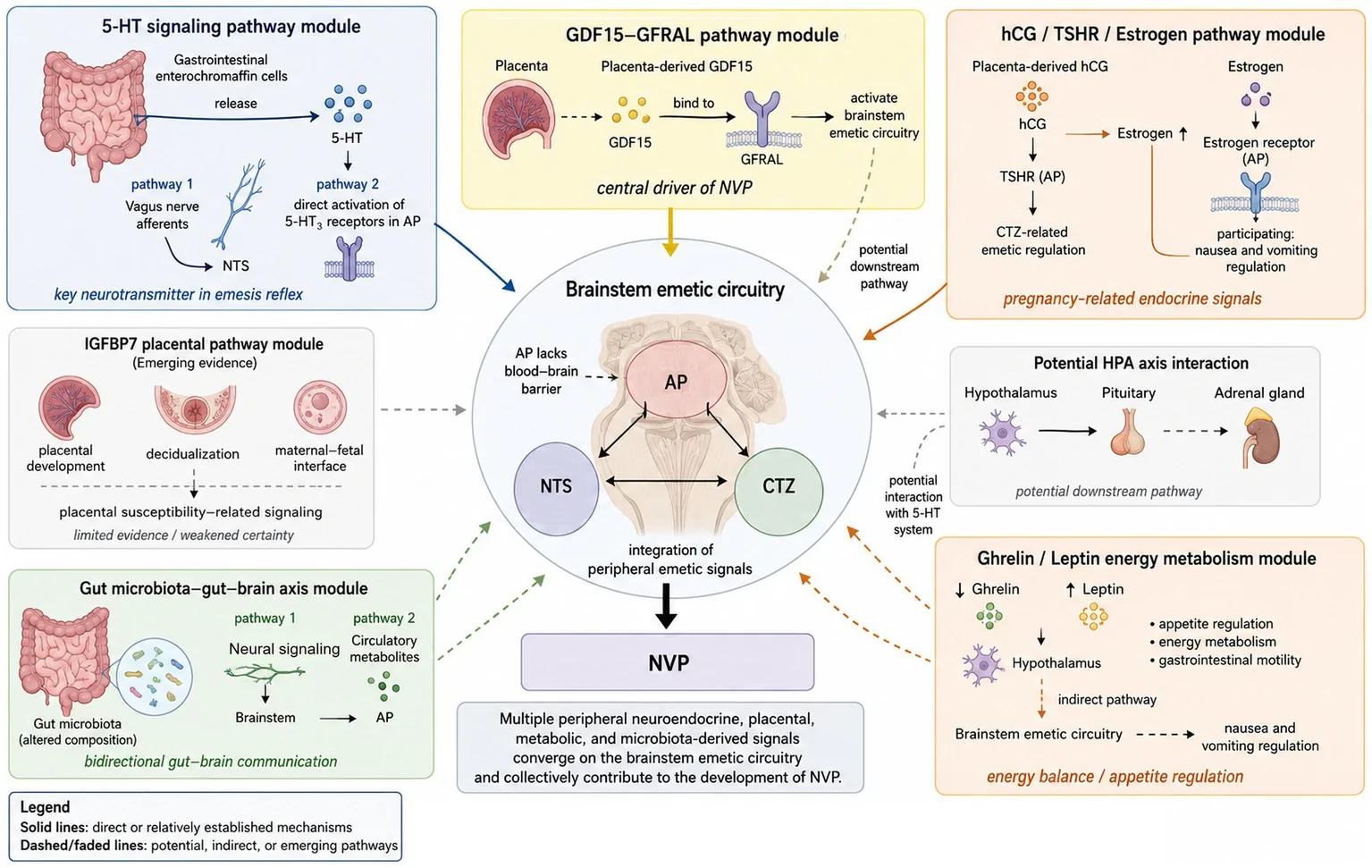
Neuroendocrine regulatory network of NVP. This mechanistic framework summarizes the major neuroendocrine, placental, metabolic, and microbiota-related pathways potentially involved in NVP pathogenesis during the first trimester. Multiple peripheral signals, including serotonergic signaling, placenta-derived GDF15, pregnancy-related endocrine signals, gut microbiota-gut-brain interactions through neural and metabolite-related pathways, appetite- and energy metabolism-related hormones, and emerging placental pathways, may converge on the brainstem emetic circuitry to contribute to NVP development. Solid lines indicate relatively established mechanisms, whereas dashed lines represent potential, indirect, or emerging pathways with weaker supporting evidence. 5-HT, 5-hydroxytryptamine; NTS, nucleus tractus solitarius; IGFBP7, insulin-like growth factor binding protein 7; AP, area postrema; GDF15, growth differentiation factor 15; GFRAL, glial cell line-derived neurotrophic factor family receptor alpha-like; CTZ, chemoreceptor trigger zone; NVP, nausea and vomiting of pregnancy; hCG, human chorionic gonadotropin; TSHR, thyroid-stimulating hormone receptor; HPA, hypothalamic–pituitary–adrenal.

Pregnancy-related changes in vitamin B6 status, including altered circulating PLP levels and increased maternal-fetal demand, may affect overall maternal nutritional status and further influence physiological regulatory processes associated with nausea and vomiting. Therefore, understanding the core mechanisms underlying NVP is important for clarifying the potential role of vitamin B6 in this condition. However, the etiology of NVP remains incompletely understood and is likely multifactorial, involving the regulation of multiple neuroendocrine axes (49). The vomiting reflex is primarily regulated by the vomiting center and the CTZ, which is located in the AP and can sense circulating chemicals and hormones because it lacks blood–brain barrier protection (50). Given this complexity, the following sections summarize potential mechanisms of NVP from the perspectives of neuroendocrine regulation, placenta-derived signaling molecules, appetite- and energy metabolism-related hormones, and the gut microbiota-gut-brain axis. These mechanisms may converge on the AP, CTZ, and related brainstem circuitry to regulate nausea and vomiting responses. See Figure 2 for details.

### 5.1 Neuroendocrine regulatory pathways

5-HT is a multifunctional monoaminergic signaling molecule mainly synthesized by enterochromaffin cells in the gastrointestinal tract; during pregnancy, placental and maternal sources can also provide 5-HT to the fetus, where it participates in fetal nervous system development (51). Previous studies have shown that circulating 5-HT levels increase significantly during pregnancy and can directly activate 5-HT₃ receptors on vagal afferent nerve terminals, transmitting signals through the vagus nerve to the nucleus tractus solitarius (NTS), or directly act on 5-HT₃ receptors in the AP, thereby jointly activating the emetic reflex center (52).

Clinical and animal studies further support a key role of 5-HT in NVP. Cengiz et al. found that plasma 5-HT levels were significantly higher in patients with HG than in women with morning sickness or asymptomatic pregnant women, and that 5-HT levels were positively correlated with HG severity (53). In a mouse study, Cui et al. found that serum 5-HT levels in late pregnancy were nearly 1,000 times higher than those in non-pregnant mice (54). Lehmann et al. reported that pregnant women carrying high-affinity 5-HT₃B receptor genotypes required higher doses of antiemetic medications, whereas high-expression 5-HT₃A subunit genotypes were associated with higher PUQE scores, suggesting that individual differences in 5-HT₃ receptor subtypes may influence NVP severity and treatment response (55).

Correspondingly, 5-HT₃ receptor antagonists have demonstrated favorable therapeutic effects in severe NVP, and ondansetron has been included as a treatment option for HG in several international guidelines (56). As another 5-HT₃ receptor antagonist, granisetron, administered via transdermal or intravenous routes, can achieve plasma concentrations comparable to those with oral administration and relieve nausea and vomiting, which may be particularly useful for patients who cannot tolerate oral treatment (57). However, current studies investigating the relationship between 5-HT and NVP still have several limitations. Existing evidence is mainly derived from case–control studies, animal models, and receptor-related genetic analyses, with differences across studies in population characteristics, disease severity, and the indicators measured. In addition, the relationship between peripheral circulating 5-HT levels and central emetic pathway activity remains unclear. Therefore, the specific regulatory mechanisms of the 5-HT system in NVP, as well as the influence of individual differences on symptom severity and treatment response, warrant further investigation.

### 5.2 Roles of placenta-derived signaling molecules

In addition to maternal neuroendocrine regulation, placenta-derived signaling molecules may provide another link between pregnancy-specific maternal-fetal interface processes and NVP. GDF15 is a stress-responsive cytokine that has been implicated in the regulation of appetite, food preference, nausea and vomiting, gastric emptying, and energy expenditure (58). A study has suggested that GDF15 may be closely associated with the development and symptom severity of NVP and is primarily secreted by the placenta (59). Its levels progressively increase throughout pregnancy (60) and decline significantly after delivery (61).

The expression of its receptor is restricted to the hindbrain, with particularly high expression in the AP (62). Accordingly, increasing evidence suggests that GDF15 may regulate neural pathways associated with NVP. Elevated circulating GDF15 levels during pregnancy may bind to its receptor, GFRAL, and subsequently activate relevant brainstem neural circuitry involved in the regulation of nausea and vomiting responses (63, 64).

Through genome-wide association studies and independent cohort validation, the Fejzo group identified significant associations between GDF15-related genetic variants and HG (65). GDF15 has also been shown to participate in the pathogenesis of familial and recurrent HG, providing new genetic insights into NVP (66). Subsequent serological analyses further confirmed an association between altered GDF15 levels and the risk of HG (67). A common coding variant in the GDF15 gene has been reported to be significantly associated with HG and proposed as a potential pathogenic variant (68). A genome-wide association study conducted in a Japanese population also suggested that GDF15-related loci may be associated with susceptibility to NVP, providing further evidence for shared genetic susceptibility across different populations (69).

In addition, pre-pregnancy exposure levels of GDF15 may alter maternal sensitivity to GDF15 and thereby influence symptom severity in NVP (59). With further research, the Cimino group found that placenta-derived GDF15 can bind to GFRAL, activate medullary neural circuits, and further stimulate the HPA axis, thereby increasing corticosterone levels (70). This finding suggests that the HPA axis may serve as an important downstream effector pathway in GDF15-mediated nausea and vomiting during pregnancy. Notably, bidirectional regulation exists between the 5-HT system and the HPA axis (71). A study by Yoo et al. suggested that the 5-HT system may participate in appetite suppression and energy intake-related processes through interactions with the HPA axis (72).

hCG is a pregnancy-specific hormone produced by placental syncytiotrophoblasts and is involved in progesterone secretion, angiogenesis, placental formation, maternal immune tolerance to pregnancy, and fetal organ differentiation and growth (73). Maternal circulating total hCG levels are associated with the severity of NVP (74, 75), and the peak concentration of hCG coincides with the peak severity of NVP symptoms, suggesting that hCG is an important contributor to NVP (76).

HCG shares structural homology with thyroid-stimulating hormone (TSH), and high levels of hCG can bind to and activate the TSH receptor (TSHR), resulting in transient gestational hyperthyroidism (77). Because TSHR is expressed in the AP, circulating hCG may directly act on TSHR in this region, activate downstream signaling pathways, and trigger the emetic reflex (78). In addition, hCG sustains the corpus luteum and promotes estrogen secretion; because estrogen receptors are highly expressed in the AP, elevated estrogen levels may contribute to nausea and vomiting responses through CTZ-related pathways (79). Moreover, hCG in combination with psychological factors is independently associated with nausea and vomiting in early pregnancy (80).

IGFBP7 is a secreted protein involved in placental development and embryo implantation-related processes, and has been proposed to be associated with genetic susceptibility to NVP (81). IGFBP7 participates in endometrial decidualization and embryo implantation during early pregnancy, is markedly upregulated after implantation, and is highly expressed in the placenta (67). Experimental evidence further supports its functional relevance, showing that IGFBP7 inhibition may disrupt decidualization by altering the uterine immune environment, thereby impairing early pregnancy maintenance (66).

Through genome-wide association studies of genetic factors related to nausea and vomiting during pregnancy, Fejzo et al. found that the chr4q12 locus was significantly associated with HG, and the nearby gene was IGFBP7, suggesting that this gene may contribute to genetic susceptibility to nausea and vomiting during pregnancy (65).

Although placenta-derived signaling molecules have been increasingly implicated in the pathophysiology of NVP, several limitations in the current evidence should be acknowledged. Existing evidence primarily derives from observational studies, genetic association analyses, and experimental models, whereas direct causal evidence in humans remains limited. In addition, the specific mechanistic pathways and potential interactions among different signaling molecules in NVP have not yet been fully elucidated and warrant further investigation.

### 5.3 Regulation by appetite- and energy balance-related hormones

Appetite- and energy balance-related hormones may also contribute to NVP by influencing gastrointestinal motility, appetite regulation, and systemic energy homeostasis. Ghrelin is a gastrointestinal peptide hormone primarily secreted by the gastric fundus and acts as an endogenous ligand for growth hormone secretagogue receptor (GHSR), regulating appetite, body weight, and energy homeostasis through central pathways involving the hypothalamus, particularly during fasting (82). Ghrelin also promotes gastric emptying and modulates gastrointestinal motility, thereby helping maintain gastrointestinal homeostasis (82). Through the gut-brain axis, ghrelin may link peripheral gastrointestinal signals with central appetite and energy-regulatory pathways, and has therefore been proposed to be associated with HG development and gestational weight changes (83). In a case–control study including 40 women with HG and 38 healthy pregnant women, serum ghrelin levels were significantly lower in the HG group, suggesting that ghrelin may be involved in HG pathogenesis and associated with dysregulated appetite and energy metabolism (84).

Leptin is a polypeptide hormone mainly secreted by adipose tissue and is also produced by the placenta during pregnancy; its levels gradually increase throughout gestation and regulate appetite, energy metabolism, systemic energy balance, and body weight through hypothalamic pathways (85). Given its role in appetite regulation and energy homeostasis during pregnancy, leptin has been investigated as a potential factor involved in NVP and HG. One clinical study reported higher serum leptin levels and an elevated leptin-to-adiponectin ratio in patients with HG, with the latter associated with disease severity, suggesting that leptin may be involved in HG development and progression (86). In addition, the study of gastrointestinal-related hormones in HG has shown that serum leptin levels are significantly elevated in patients with HG and are accompanied by changes in ghrelin and other hormones, suggesting that leptin may act together with gastrointestinal hormones in the pathogenesis (87). Another study further found that serum leptin levels were elevated in patients with HG and were associated with inflammatory markers and oxidative stress status, suggesting that leptin may contribute to HG development by participating in inflammatory and oxidative stress-related processes (88). However, studies investigating the roles of appetite- and energy metabolism-related hormones in NVP still have several limitations. Most available studies are case–control studies with relatively small sample sizes, and differences exist across studies in population characteristics, disease definitions, and measured indicators. For example, some studies primarily focused on changes in ghrelin or leptin levels, whereas others further examined the leptin/adiponectin ratio, oxidative stress, or *Helicobacter pylori*-related factors. Therefore, the specific roles of these hormones in the pathogenesis of HG and their causal relationships remain to be further clarified.

### 5.4 Gut microbiota-gut-brain axis interaction mechanisms

The gut microbiota-gut-brain axis may provide another pathway through which gastrointestinal and neuroendocrine signals contribute to NVP and HG. The gut microbiota can communicate bidirectionally with the central nervous system through the gut-brain axis, a process involving multiple neural, endocrine, and immune signaling pathways, thereby potentially influencing gastrointestinal symptoms such as nausea and vomiting (89).

A nested case–control study showed that more severe nausea and vomiting in early pregnancy was associated with lower fecal microbiota richness and diversity, as well as altered abundances of specific bacterial taxa, including a negative association between Lactobacillaceae and symptom severity and a positive association between *Bifidobacterium dentium* and more severe symptoms (90). Another study found that patients with HG exhibited significantly increased gut microbial *α*-diversity and greater abundance of bacterial taxa, including Bacteroidaceae, Firmicutes, Clostridia, and Betaproteobacteria (91). In addition, fecal microbiota analyses have shown that Clostridium spp. are increased. In contrast, Bifidobacterium spp. are decreased in patients with HG (92).

Further Mendelian randomization studies have suggested potential causal associations between gut microbial taxa and HG risk, with Defluviitaleaceae UCG011, Ruminococcus, Turicibacter, and Verrucomicrobiota associated with increased HG risk, whereas Coprococcus2 may exert a protective effect (93). Although increasing evidence suggests that the gut microbiota-gut-brain axis may be involved in the development of NVP, current findings remain preliminary and somewhat inconsistent. Some studies have reported alterations in gut microbiota composition and diversity in women with NVP, which appear to be associated with symptom severity; however, findings regarding microbial diversity remain inconsistent, with some studies reporting reduced microbial richness and others reporting increased *α*-diversity. Recent Mendelian randomization analyses have further suggested potential causal associations between specific microbial taxa and HG risk, although the underlying biological mechanisms and causal relationships remain to be further clarified.

Collectively, studies on neurotransmitter regulation, placenta-derived factors, hormonal alterations, and the gut-brain axis suggest that NVP development may involve a complex neuroendocrine and metabolic regulatory network. These mechanistic insights may also provide a potential biological rationale for the clinical application of vitamin B6 as a foundational treatment for NVP.

## 6 Clinical application of vitamin B6 in the management of NVP

At present, obstetric and gynecologic guidelines from multiple countries recommend exogenous vitamin B6 supplementation as a basic pharmacologic treatment for NVP. The American College of Obstetricians and Gynecologists states that vitamin B6 alone or in combination with doxylamine is safe and effective for the treatment of NVP and should be used as a first-line pharmacologic option (94). The Royal College of Obstetricians and Gynecologists states that the prolonged-release combination of doxylamine and vitamin B6 is the only drug approved in the United Kingdom for the treatment of NVP and is suitable for mild-to-moderate cases (95). The French National College of Obstetricians and Gynecologists indicates that vitamin B6 may be used for mild NVP, and that the doxylamine-pyridoxine combination is associated with the fewest adverse effects among pharmacologic treatments used in early pregnancy (96). Although recommendations vary across guidelines, particularly for pyridoxine monotherapy, vitamin B6 alone or combined with doxylamine remains an important early pharmacological option for mild-to-moderate NVP.

### 6.1 Clinical efficacy and dosing regimens

From a clinical perspective, multiple systematic reviews and meta-analyses have confirmed that vitamin B6 supplementation can effectively reduce symptoms of nausea and vomiting during pregnancy and has a favorable safety profile (27). A secondary analysis of a multicenter randomized controlled trial showed that higher serum PLP levels in early pregnancy were associated with faster NVP symptom relief, with women in the highest PLP group having about a 46% lower risk of persistent symptoms than those in the lowest group (97). These findings suggest that PLP may help predict symptom improvement.

In another study, Wibowo et al. randomly assigned 60 pregnant women to high-dose (10 mg) or low-dose (1.28 mg) vitamin B6 for 2 weeks and found that supplementation increased plasma vitamin B6 concentrations and improved PUQE scores (98). Although score reduction was greater in the high-dose group, the difference had limited clinical impact on symptom severity. Pope et al. conducted a 1-week intervention comparing pyridoxine monotherapy with doxylamine-pyridoxine combination therapy and found that the combination group had significantly lower PUQE scores than the pyridoxine-only group, with fewer women remaining in the moderate-to-severe category during follow-up (99). Koren et al. reported that prolonged-release doxylamine-pyridoxine improved NVP symptoms on days 3, 4, and 5, and the therapeutic effect persisted through day 14 (100).

Using *in vitro* dissolution testing, human pharmacokinetic studies, and computer simulation, Cariban® prolonged-release capsules (10 mg doxylamine + 10 mg pyridoxine) were shown to provide early, gradual, and sustained release, and the clinically recommended “1–1-2” regimen, defined as one capsule in the morning, one in the mid-afternoon, and two at bedtime, achieved the most stable and prolonged plasma drug concentrations, supporting around-the-clock symptom control (101). Another dual-release doxylamine-pyridoxine formulation is a multilayer modified-release tablet containing an enteric-coated core and an immediate-release coating, with a total dose of 20 mg doxylamine and 20 mg pyridoxine; this design reduces dosing frequency from three times daily to twice daily while providing both immediate and sustained efficacy and improving patient adherence (102). These findings suggest that the formulation choice should be tailored to symptom severity and patient compliance. Currently, vitamin B6 alone at a dose of <100 mg/day is recommended as initial treatment for NVP (94). In contrast, the standard dose of vitamin B6 combined with doxylamine is generally 10 mg doxylamine + 10 mg pyridoxine or 20 mg doxylamine + 20 mg pyridoxine, with a maximum daily dose of 40 mg doxylamine + 40 mg pyridoxine (95). See Table 1 for details.

**Table 1:** Clinical evidence on efficacy, dosing, and safety of vitamin B6 in NVP.

Author (Year)	Study type	Intervention/dose	Duration	Main findings	Safety/adverse events
Wibowo et al. (2012) (98)	Clinical study	Pyridoxine 10 mg/day (5 mg twice daily) vs. 1.28 mg/day (0.64 mg twice daily)	2 weeks	The higher-dose group showed a greater reduction in PUQE scores and a more pronounced increase in plasma vitamin B6 levels; however, the authors suggested that this difference may not be clinically meaningful in symptom severity.	No significant serious adverse events were reported in the abstract; the study primarily focused on symptom improvement and plasma B6 levels.
Pope et al. (2015) (99)	Matched cohort study	Pyridoxine monotherapy (mean 99.1 mg/day, up to approximately 200 mg/day) vs. doxylamine-pyridoxine combination	1 week	Combination therapy demonstrated superior efficacy compared with pyridoxine alone, particularly in moderate-to-severe NVP (PUQE score improvement: 2.6 vs. 0.4).	The study primarily evaluated efficacy; no new safety signals were reported in the abstract.
RCOG Guideline No. 69 (2016) (95)	Guideline	Pyridoxine monotherapy is not recommended for NVP	–	Due to limited evidence of efficacy, pyridoxine alone is not recommended for the treatment of NVP.	No specific safety concerns were emphasized; recommendations were based on the strength of the evidence rather than toxicity.
Koren et al. (2016) (100)	Randomized controlled trial (secondary analysis)	Doxylamine 10 mg + pyridoxine 10 mg per tablet; 2–4 tablets/day	14 days (early response observed within 3–5 days)	Compared with placebo, symptoms improved significantly, with the greatest effect observed around Day 4 (approximately 40% reduction in PUQE score).	No new safety concerns were identified; this secondary analysis primarily focused on early efficacy.
ACOG Committee on Practice Bulletins-Obstetrics (2018) (94)	Guideline	Pyridoxine monotherapy (10–25 mg, 3–4 times daily) or in combination with doxylamine (12.5 mg) as first-line therapy	–	Based on multiple clinical studies, pyridoxine alone or in combination is effective for NVP; early intervention may prevent progression to HG.	Extensive clinical use supports its safety, with no evidence of teratogenicity; combination therapy may be associated with mild central nervous system effects such as drowsiness and fatigue.
Jayawardena et al. (2023) (27)	Systematic review and meta-analysis	Pyridoxine monotherapy (0.64–50 mg twice daily) or combination therapy (10–50 mg)	3–60 days	Overall improvement in NVP symptoms; significant benefits observed in Rhodes score (MD = 0.78, *p* = 0.003) and PUQE score (MD = 0.75, *p* = 0.002).	No increased risk of adverse pregnancy outcomes or congenital anomalies; moderate heterogeneity was observed (I^2^ = 57%).
Saz-Leal et al. (2023) (101)	Pharmacokinetic study	Modified-release doxylamine/pyridoxine (10/10 mg); titrated from 0–0–2 to 1–1–2 dosing regimen	–	Sustained drug release with stable plasma exposure; the 1–1–2 regimen maintains therapeutic levels over 24 h.	No treatment-emergent adverse events were reported; the study provided pharmacokinetic support for the recommended dosing regimen.
Schleußner et al. (2024) (102)	Review	Dual-release doxylamine-pyridoxine formulation (20 mg/20 mg per tablet); 1–2 tablets/day (morning and evening)	–	The dual-release formulation provides both immediate and sustained drug release, allowing twice-daily dosing and supporting symptom control.	The formulation was reported to be well tolerated; available evidence does not indicate teratogenicity of pyridoxine at doses ≤200 mg/day.
Gao et al. (2026) (97)	Secondary analysis	Endogenous PLP levels (no intervention)	–	Higher first-trimester serum PLP levels were associated with faster natural resolution of NVP (highest vs. lowest quartile: 46% reduction in the risk of symptom persistence).	Not a safety study; supports a biological association between vitamin B6 status and NVP progression.

Overall, current clinical evidence supports vitamin B6, particularly in combination with doxylamine, as an early pharmacological option for mild-to-moderate NVP. Modified-release formulations may improve pharmacokinetic stability and symptom control. However, existing studies remain heterogeneous in dosing regimens, intervention duration, outcome measures, and study design, and evidence remains limited for severe HG, long-term efficacy, and pyridoxine monotherapy. In addition, guideline recommendations for pyridoxine monotherapy are inconsistent: ACOG supports vitamin B6 alone or with doxylamine as first-line treatment, whereas RCOG does not recommend pyridoxine monotherapy. Therefore, further high-quality randomized controlled trials are needed to better define target populations, dosing strategies, and clinical efficacy.

### 6.2 Safety evaluation and prevention of adverse events

Medication safety in NVP treatment must take into account both maternal pregnancy outcomes and fetal development. Given the widespread use of vitamin B6 supplementation, its safety is particularly important. Extensive clinical experience has demonstrated that the doxylamine-pyridoxine combination is generally well tolerated by pregnant women and has a favorable fetal safety profile, with no increase in the incidence of adverse events such as central nervous system depression, gastrointestinal complications, or cardiovascular involvement (103, 104). However, prolonged or high-dose vitamin B6 exposure has been associated with potential neurotoxicity, including peripheral neuropathy. Some evidence also suggests that high-dose use (≥50 mg/day) in early pregnancy may be associated with miscarriage, intrauterine death, and congenital malformations, although a definite causal relationship has not been established (105). Therefore, careful dose selection and individualized assessment may be warranted in clinical practice, with efforts to avoid prolonged high-dose exposure and to monitor neurologic symptoms and fetal development appropriately.

A large cohort study from Quebec reported that first-trimester exposure to doxylamine-pyridoxine was associated with an increased risk of major congenital malformations overall and with increased risks of spina bifida, neurologic defects, and musculoskeletal defects (106). These findings are inconsistent with previous observational studies. Subsequently, Biffi et al. performed a meta-analysis and argued that the study may have been affected by biases, including false-positive case selection, exposure misclassification, and outcome misclassification (107). Therefore, its conclusions should be interpreted with caution, and further scrutiny of existing databases is warranted before drawing definitive safety conclusions. A randomized, placebo-controlled trial demonstrated that doxylamine-pyridoxine combination therapy did not increase the risk of adverse pregnancy outcomes compared with placebo (108). A systematic review and meta-analysis specifically evaluating vitamin B6 supplementation for NVP concluded that pyridoxine alone or in combination with active compounds may offer potential benefits for women with NVP (27). However, the included studies were limited by inconsistent outcome measures and several confounding factors. Accordingly, future research should focus on better-designed clinical trials to provide higher-quality evidence.

Overall, most studies and international guidelines suggest that vitamin B6, particularly when combined with doxylamine, has a favorable safety profile for NVP when used within recommended therapeutic doses. However, inconsistent observational findings and potential risks associated with prolonged high-dose exposure indicate that its safety profile still requires careful interpretation. These discrepancies may be related to differences in study design, dose exposure, exposure misclassification, and inadequate control of confounding factors. Therefore, future large-scale studies with improved exposure assessment and long-term maternal-fetal follow-up are needed to further clarify its safety profile.

## 7 Core mechanisms by which vitamin B6 regulates NVP

Although vitamin B6 has been widely used in the clinical management of NVP and is recommended by multiple international guidelines, its underlying biological mechanisms remain incompletely understood. Emerging evidence suggests that vitamin B6 may regulate nausea and vomiting through multiple pathways by maintaining neurotransmitter metabolic homeostasis and neuro-metabolic balance (109–111).

With respect to neurotransmitter regulation, PLP may influence nausea- and vomiting-related pathways by acting as an essential cofactor for aromatic L-amino acid decarboxylase, which catalyzes the conversion of 5-hydroxytryptophan to 5-HT (112). Increased peripheral 5-HT release from enterochromaffin cells in the gastrointestinal tract can activate vagal afferent pathways, transmitting signals to the NTS and CTZ in the medulla and ultimately contributing to nausea and vomiting (113). PLP may also influence NVP-related neural regulation through GABA metabolism. As discussed above, GABA synthesis depends on PLP-dependent enzymatic activity, and pyridoxine has been shown to affect GAD and other enzymes involved in GABA synthesis and degradation (114). Given the inhibitory role of GABA in central neurotransmission, altered vitamin B6 status, particularly reduced PLP availability, may influence neural excitability in pathways relevant to nausea and vomiting. As pregnancy may alter maternal vitamin B6 metabolism, reduced PLP availability could impair key decarboxylation reactions involved in 5-HT and GABA metabolism, thereby disrupting neurotransmitter homeostasis and increasing susceptibility to nausea- and vomiting-related stimuli. However, direct mechanistic evidence in human pregnancy remains limited and requires further validation.

## 8 Limitations and future research directions

Although existing studies support the potential clinical value of vitamin B6 in NVP management, particularly for mild-to-moderate symptoms, several limitations remain. First, substantial heterogeneity exists across studies in dosage regimens, formulations, intervention duration, outcome measures, and study populations, which limits comparability and generalizability. Many studies also have small sample sizes, short follow-up periods, or differing study designs. Second, current clinical evidence focuses mainly on mild-to-moderate NVP, whereas evidence for HG remains limited. Optimal dosing regimens, long-term efficacy, and combination treatment strategies for severe disease have not been fully established.

Mechanistic evidence is also limited. Most available data are derived from basic experiments, animal models, or indirect clinical observations, and direct clinical evidence linking pregnancy-related PLP levels to NVP severity remains insufficient. In addition, interindividual differences in response to vitamin B6, inconsistencies in guideline recommendations for pyridoxine monotherapy, and the long-term maternal-fetal safety of prolonged high-dose supplementation require further clarification. Future studies should include large-scale, high-quality randomized controlled trials with long-term follow-up to optimize dosing and treatment strategies, clarify vitamin B6-related mechanisms in NVP, and support more individualized clinical management.

## 9 Conclusion

NVP is a common but mechanistically complex condition in early pregnancy, involving multiple neuroendocrine and metabolic pathways, including neurotransmitter regulation, placenta-derived signaling molecules, energy metabolism-related hormones, and the gut-brain axis. Pregnancy-related changes in maternal vitamin B6 status may interact with these pathways and contribute to symptom development and improvement. As a first-line treatment for mild-to-moderate NVP, vitamin B6 has favorable clinical utility, particularly when combined with doxylamine. Mechanistically, its active form, PLP, may influence gastrointestinal-vagal-brainstem emetic pathways by modulating neurotransmitter metabolism, including 5-HT and GABA. However, direct clinical evidence supporting these mechanisms remains limited. Future studies integrating PLP and neurotransmitter-related biomarkers with high-quality clinical investigations are needed to clarify the mechanistic role of vitamin B6 in NVP and optimize individualized treatment strategies.
